# Tauopathy in veterans with long-term posttraumatic stress disorder and traumatic brain injury

**DOI:** 10.1007/s00259-018-4241-7

**Published:** 2019-01-07

**Authors:** Abdalla Z. Mohamed, Paul Cumming, Jürgen Götz, Fatima Nasrallah, for the Department of Defense Alzheimer’s Disease Neuroimaging Initiative

**Affiliations:** 1 Queensland Brain Institute, University of Queensland, Building 79, Upland Road, Saint Lucia, Brisbane, Queensland 4072 Australia; 2 School of Psychology and Counselling and IHBI, Queensland University of Technology, Brisbane, QLD 4059 Australia; 3 QIMR-Berghofer Institute, Brisbane, QLD 4006 Australia; 4 Clem Jones Centre for Ageing Dementia Research (CJCADR), Queensland Brain Institute, The University of Queensland, Brisbane, QLD 4072 Australia

**Keywords:** Traumatic brain injury, Posttraumatic stress disorder, Alzheimer’s disease, Tau, Positron emission tomography, US Department of Defense Alzheimer’s Disease Neuroimaging Initiative

## Abstract

**Purpose:**

Traumatic brain injury (TBI) and posttraumatic stress disorder (PTSD) have emerged as independent risk factors for an earlier onset of Alzheimer’s disease (AD), although the pathophysiology underlying this risk is unclear. Postmortem studies have revealed extensive cerebral accumulation of tau following multiple and single TBI incidents. We hypothesized that a history of TBI and/or PTSD may induce an AD-like pattern of tau accumulation in the brain of nondemented war veterans.

**Methods:**

Vietnam War veterans (mean age 71.4 years) with a history of war-related TBI and/or PTSD underwent [^18^F]AV145 PET as part of the US Department of Defense Alzheimer’s Disease Neuroimaging Initiative. Subjects were classified into the following four groups: healthy controls (*n* = 21), TBI (*n* = 10), PTSD (*n* = 32), and TBI+PTSD (*n* = 17). [^18^F]AV1451 reference tissue-normalized standardized uptake value (SUVr) maps, scaled to the cerebellar grey matter, were tested for differences in tau accumulation between groups using voxel-wise and region of interest approaches, and the SUVr results were correlated with neuropsychological test scores.

**Results:**

Compared to healthy controls, all groups showed widespread tau accumulation in neocortical regions overlapping with typical and atypical patterns of AD-like tau distribution. The TBI group showed higher tau accumulation than the other clinical groups. The extent of tauopathy was positively correlated with the neuropsychological deficit scores in the TBI+PTSD and PTSD groups.

**Conclusion:**

A history of TBI and/or PTSD may manifest in neurocognitive deficits in association with increased tau deposition in the brain of nondemented war veterans decades after their trauma. Further investigation is required to establish the burden of increased risk of dementia imparted by earlier TBI and/or PTSD.

**Electronic supplementary material:**

The online version of this article (10.1007/s00259-018-4241-7) contains supplementary material, which is available to authorized users.

## Introduction

Alzheimer’s disease (AD) is the most common form of dementia in the elderly, leading to a progressive deterioration of memory and spatial cognition, along with other cognitive impairments [1]. AD pathology is characterized by the aggregation of amyloid-β and phosphorylated tau [2–4], and tau deposition is particularly associated with progression of clinical symptoms [2]. It is increasingly recognized that traumatic brain injury (TBI) and posttraumatic stress disorder (PTSD) increase the risk of cognitive decline and dementia [5, 6], suggesting a link with AD. In addition, there is considerable comorbidity of PTSD with TBI in both civilian and military settings [7–9], which raises the possibility of synergistic effects favouring the risk of dementia.

A retrospective cohort study by Yaffe et al. showed that veterans with PTSD have a twofold higher risk of developing dementia than veterans without PTSD [10]. In addition, a systematic review revealed an association between TBI and the development of AD with an odds ratio of 2.3 [11]. These associations imply that the two conditions may interact by increasing the risk of neurodegeneration and dementia. Indeed, several neuroimaging studies have shown overlapping patterns of brain volume loss in TBI, PTSD and AD [12–14]. Post-mortem investigations have shown intraneuronal tau accumulation after a single TBI incident [15] and in subjects with multiple TBI events suffering from chronic traumatic encephalopathy (CTE) [16]. PET with [^18^F]AV1451 [17] and other tau ligands [18] has recently been used to detect tau deposits in the brain of living AD patients. There is a single report in abstract form of tauopathy in the cerebral cortex of living veterans with PTSD [19].

According to the National Institute on Aging and the Alzheimer's Association (NIA-AA) research framework, AD is defined by the presence of both amyloid-β and pathological tau deposits. However, when amyloid deposition is accompanied by primary age-related tauopathy, the disorder should properly be designated as “Alzheimer’s pathological change” which could be considered as an early presentation of the “Alzheimer’s continuum” [20]. Tau PET is a new in vivo molecular imaging modality used to investigate the progression of tauopathy in the brain, and has been correlated with the Braak neurofibrillary tangle (NFT) stages as defined post mortem [21]. Indeed, Schwarz et al. used [^18^F]AV1451 PET to identify Braak stages that represent the well-defined neuroanatomical signature of tau pathology in typical AD [22]. Furthermore, elevated tau binding on PET has been shown to be associated with amyloid positivity and cognitive impairment in both normal ageing and dementia [20, 22–24].

Inspired by this background, we analysed tau PET data that had been acquired using [^18^F]AV1451 PET from the Alzheimer's Disease Neuroimaging Initiative-Department of Defense (ADNI-DOD) study of nondemented Vietnam War veterans suffering from service-related TBI, PTSD, and comorbid TBI with PTSD. Using parametric mapping procedures, we evaluated tau deposition in cohorts with TBI and/or PTSD compared with healthy veterans, and addressed the relationship between the individual tau burden and cognitive test scores. In addition, we investigated tau pathology in relation to amyloid PET findings and to the histopathological Braak stages, which were defined using the criteria of Schwarz et al. [22].

## Materials and methods

### Study design

All data were obtained from the ADNI-DOD which is a multimodal (MRI, PET and neuropsychological assessment), nonrandomized study that recruited Vietnam War veterans selected from the Department of Veterans Affairs compensation and pension records, investigating TBI and/or PTSD as potential risk factors for the development of AD. ADNI-DOD is part of the ADNI project launched in 2003 as a public/private partnership led by Principal Investigator Michael W. Weiner, MD. All participants signed a consent form, and the use of deidentified data was approved by the Human Research Ethics Committee of the University of Queensland, Australia (IRB number 2017000630).

[^18^F]AV1451 PET for tau imaging had been performed in a total of 99 subjects as part of the ADNI-DOD study, and 81 of these subjects had their T1-weighted structural MRI data available at the time of this research. Data from one female participant was excluded to avoid gender effects, leaving a total of 80 datasets from male Vietnam War veterans of mean age 71.4 ± 5.1 years. PET with [^18^F]AV45 for amyloid imaging had also been performed in all 80 subjects. Subjects were classified into the following four groups: healthy controls (*n* = 21), moderate/severe TBI (*n* = 10), PTSD (*n* = 32), and TBI with PTSD (TBI+PTSD, *n* = 17). All subjects’ clinical categories were identified from the “VAELG.csv” file provided by the ADNI-DOD administration. In addition, subjects with mild cognitive impairment (MCI) were identified by ADNI-DOD based on cognitive test scores. The TBI subjects had a documented history of moderate-to-severe nonpenetrating TBI during their military service. PTSD subjects were identified using the clinician-administered PTSD scale (CAPS) within DSM-IV (CAPS score >40).

In addition to imaging, all participants completed several neuropsychological questionnaires, including Everyday Cognition (ECog), Clinical Dementia Rating (CDR), Mini-Mental State Examination (MMSE), Montreal Cognitive Assessment (MOCA), Alzheimer’s Disease Assessment Scale–Cognitive (ADAS-Cog), Geriatric Depression Scale, Functional Assessment Questionnaire, Combat Exposure Scale, and the Armed Forces Qualification Test (AFQT). All participants were also assessed using a battery of neuropsychological tests including the Clock Drawing Test, the Rey Auditory Verbal Learning Test, the Category Fluency Test, the Trail Making Test, the Boston Naming Test, and the American National Adult Reading Test. The ECog ratings were reported by the participants that cover multiple cognitive domains, including language, memory, visual spatial ability, and executive function, including planning, organization, and divided attention. The ECog questionnaire contains 39 items, which are rated on a four-point scale: 1 = better or no change compared with 10 years earlier, 2 = questionable/occasionally worse, 3 = consistently a little worse, 4 = consistently much worse, and subjects can respond with “9” if they wish to indicate “I don’t know.” The score of each category was calculated as the average of the answered questions in each subcategory and the total ECog score as the mean of all answered questions in all categories.

### MRI/PET image acquisition and processing

PET tau imaging was performed with [^18^F]AV1451. Data acquisition procedures were standardized across all ADNI sites (information can be found at: http://adni.loni.usc.edu/wp-content/uploads/2015/02/01_DOD-ADNI_Tau-Addendum-Protocol_23Oct2014.pdf). Data were preprocessed and analysed as described in our previous paper [25] using the FMRIB software library (FSL 5.0.9). MRI images were corrected for intensity inhomogeneity, skull-stripped, and segmented using the RECON-ALL [26] from Freesurfer. Structural data were then resampled to an isotropic resolution of 1.5 mm and normalized to the Montreal Neurological Institute (MNI) structural template nonlinearly using FSL-FNIRT [27].

The preprocessed data were downloaded from ADNI-DOD (http://adni.loni.usc.edu/methods/pet-analysis-method/pet-analysis/). The four sequential emission frames were coregistered, and standardized uptake values (SUV) were calculated and averaged. SUV maps were intensity-normalized and spatially smoothed using a scanner-specific filter function to generate SUV maps with a uniform isotropic resolution of 8 mm full-width at half-maximum. The SUV maps were skull-stripped using FSL-BET and linearly coregistered to each individual’s T1-weighted image using FSL-FLIRT. Each individual’s SUV map was scaled to the mean intensity in a cerebellar grey matter template to generate reference tissue-normalized standardized uptake (SUVr) maps [28] in the native (individual) space. Finally, [^18^F]AV1451 SUVr maps were spatially normalized to the MNI template using the transformation matrix and warp calculated for T1 structural MR-to-MNI registration.

To assess amyloid positivity, SUV maps of the amyloid [^18^F]AV45 PET tracer from the same subjects were downloaded. The acquisition parameters were as described previously [25]. The [^18^F]AV45 SUV maps were coregistered to individual T1-weighted MR images in native space using FSL-Flirt, and amyloid-PET SUVr values were calculated using the whole cerebellum as the reference region [22]. To identify amyloid-positive subjects, a global SUVr score was calculated which was the mean SUVr in the whole cerebral cortex, where SUVr >1.1 was deemed as amyloid-positive.

### Regions of interest and algorithm for estimating Braak staging using [^18^F]AV1451 SUVr

Braak staging is based on the characteristic progression of tau pathology starting in the medial temporal lobe and eventually encompassing the neocortex as revealed by post-mortem examination. We applied methods developed by Schwarz et al. [22], whose algorithm scores Braak staging noninvasively using [^18^F]AV1451 SUVr measured in the entorhinal cortex, hippocampus, superior and middle temporal gyri (STG, MTG), fusiform cortex, lingual gyrus (BA17), and pericalcarine visual cortex (V1+V2+V3). Whereas Schwarz et al. defined regions of interest (ROIs) in MNI space based on 2-mm isotropic voxels lying close to slices of the histological Braak staging protocol [29], we defined the same ROIs in the individual’s native space after Free-surfer segmentation. We then calculated the mean [^18^F]AV1451 SUVr in bilateral ROIs, from which we assigned the Braak stage using the staging algorithm described by Schwarz et al. [22], with visual confirmation from the SUVr maps. The final Braak stage was defined as the highest score between the two hemispheres.

### Statistical analysis

To investigate tau accumulation associated with a history of TBI and/or PTSD, the three clinical groups were compared to the healthy control group using voxel-based approaches encompassing all brain voxels of the tau PET SUVr images, using a nonparametric permutation test (FSL-randomise) with 5,000 permutations, with correction for multiple comparisons using false discovery rate (FDR) (*p* < 0.05). All analyses were corrected for ApoE4 status, age, MCI status (for confirmed cases) and hypertension. We investigated the correlation between tau accumulation and the ADAS-Cog score, ECog total score, and CDR score, as well as cerebral total amyloid. These correlations were calculated using a multilinear regression performed using (FSL-GLM) that generated Pearson correlation maps, with FDR correction for multiple comparisons (*p* < 0.05).

Statistical analyses were performed with R-studio, version 3.3.1 ® Foundation for Statistical Computing, Vienna, Austria). Differences in neuropsychological assessment measures and ROI-based SUVr values between groups were evaluated using the Kruskal–Wallis test, with the significance level set at *p* < 0.05 persisting after Bonferroni correction for multiple comparisons (*n* = 6). To investigate tau distribution in the four great lobes of the cortex, each individual’s mean regional [^18^F]AV1451 SUVr values in the frontal, cingulate, parietal, and temporal lobes were extracted.

## Results

### Clinical outcome in TBI and/or PTSD groups

**Fig. 1:**
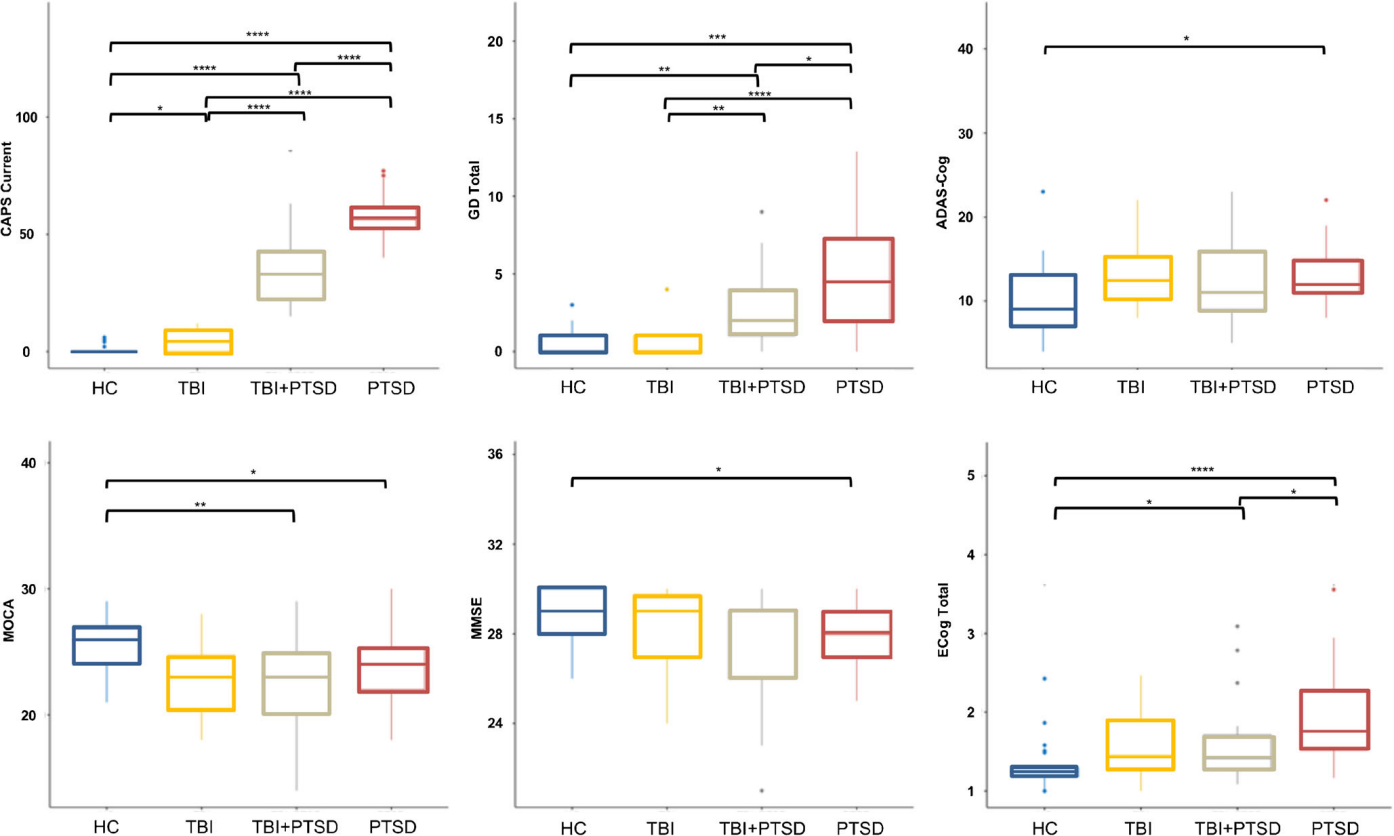
Differences in neuropsychological test scores between groups. *CAPS Current* clinician-administered PTSD scale current score, *GD Total* Geriatric Depression Scale, *ADAS-Cog* Alzheimer’s Disease Assessment Scale–Cognitive, *MOCA* Montreal Cognitive Assessment, *MMSE* Mini-Mental State Examination, *ECog* Everyday Cognition, *TBI* traumatic brain injury, *PTSD* posttraumatic stress disorder, *TBI+PTSD* TBI subjects who developed PTSD. *p < 0.05, **p < 0.01, ***p < 0.001, ****p < 0.0001

In this cross-sectional study, cognitive function in groups of veterans with a history of TBI and/or PTSD was investigated. The four subject groups were healthy controls (age 74.3 ± 7.2 years, mean ± standard deviation), TBI (72.6 ± 6.8 years), PTSD (70 ± 2.7 years), and TBI+PTSD (69.9 ± 2.5 years; Table 1). Overall, the neuropsychological test results suggested that cognitive deficits were more pronounced in the PTSD and TBI+PTSD groups than in the TBI or healthy control groups, without any subject being diagnosed with AD by any test (Fig. 1, Table 1).

**Table 1:** Demographic characteristics and neuropsychological performance of the subjects

		Group				*p* values		
		Healthy controls	TBI	TBI+PTSD	PTSD	PTSD vs. controls	TBI vs. controls	TBI+PTSD vs. controls
Demographic	Number of subjects	21	10	17	32			
	Age (years), mean (SD)	74.29 (7.2)	72.6 (6.82)	69.88 (2.5)	70 (2.72)			
	Years of education, mean (SD)	15.57 (2.5)	16.2 (1.99)	14.47 (2.48)	15.03 (2.63)			
Clinical	ApoE4 status (+/−), *n*/*n*^a^	5/16	2/6	4/7	6/23			
	Mild cognitive impairment, *n*	0	4	3	8			
	Amyloid status (+/−), *n*/*n*	8/13	7/3	9/8	18/14			
	Hypertension, *n*	12	20	5	13			
Clinician-administered PTSD scale scores	Current	0.85 (1.87)	4.67 (4.92)	36.29 (18.63)	57.72 (9.74)	<0.001		<0.001
	Life	3.45 (6.3)	13.33 (10.26)	53.76 (18.91)	77.81 (19.5)	<0.001		<0.001
Neuropsychological questionnaires	Montreal Cognitive Assessment	25.52 (2.25)	23 (3.43)	22.12 (3.66)	23.59 (2.82)	<0.05		<0.01
	MMSE	28.86 (1.06)	28.1 (2.02)	27.41 (2.62)	28.03 (1.49)	<0.05		
	ADAS–Cogitive	10.19 (4.65)	13.3 (4.74)	12.82 (6.04)	13.09 (3.31)	<0.05		
	Clinical Dementia Rating	0 (0)	0.2 (0.26)	0.18 (0.25)	0.16 (0.39)	<0.05		
	Combat Exposure Score	13.05 (5.25)	14.89 (7.9)	19.5 (6.41)	20.58 (7.45)	<0.01		<0.05
	Armed Forces Qualification Test percentile	57.68 (22.46)	58 (24.99)	45.57 (25.79)	49.7 (19.52)			
	Functional Assessment Questionnaire total	0.11 (0.33)	3.4 (5.98)	3 (4.52)	2.82 (4.22)	<0.05		
	Geriatric Depression Scale	0.81 (0.87)	0.7 (1.25)	2.71 (2.52)	4.91 (3.6)	<0.001		<0.05
	ECog Memory	1.6 (0.5)	1.95 (0.73)	2.09 (0.75)	2.43 (0.7)	<0.001		
	ECog Language	1.41 (0.56)	1.52 (0.57)	1.85 (0.80)	2.13 (0.69)	<0.001		
	ECog Spatial Visual	1.12 (0.21)	1.17 (0.36)	1.29 (0.51)	1.46 (0.61)	<0.05		
	ECog Plan	1.19 (0.23)	1.42 (0.54)	1.50 (0.73)	1.65 (0.71)	<0.05		
	ECog Organize	1.23 (0.35)	1.76 (0.58)	1.45 (0.9)	1.86 (0.85)	<0.01	<0.05	
	ECog Divide Attention	1.40 (0.47)	1.70 (0.66)	1.63 (0.9)	2.14 (0.66)	<0.001		
	ECog total	1.33 (0.32)	1.59 (0.47)	1.63 (0.58)	1.95 (0.59)	<0.001		<0.05
Neuropsychological battery	Rey Auditory Verbal Learning Test	12.62 (2.09)	12.3 (1.95)	12.12 (2.93)	12.44 (2.47)			
	Clock Drawing Test	4.48 (0.75)	4.2 (1.32)	4.18 (0.88)	4.38 (0.79)			
	Clock Copy Test	4.95 (0.22)	4.9 (0.32)	4.35 (0.61)	4.69 (0.47)			<0.001
	Category Fluency Test	22.95 (5.61)	20 (5.66)	19.76 (5.08)	18.63 (4.55)	<0.05		
	Trail Making Test – Part A	34.76 (9.16)	40.1 (15.41)	43 (30.98)	35.84 (8.27)			
	Trail Making Test – Part B	78.24 (19.99)	105.7 (47.06)	114.59 (78.99)	98.06 (41.33)			
	American National Adult Reading Test	10.76 (7.44)	13.1 (8.56)	21 (11.95)	16.84 (7.69)	<0.05		<0.01
	Boston Naming Test	28.9 (1.04)	28 (1.33)	28.12 (2.09)	27.88 (2.15)			

*ADAS* Alzheimer’s Disease Assessment Scale, *ECog* Everyday Cognition, *MMSE* Mini-Mental State Examination

^a^Numbers of subjects with available APOE4 status: healthy control group, 21; TBI group, 8; TBI+PTSD group, 11; PTSD group, 29

According to the information provided by ADNI-DOD, subjects with memory deficits were identified by applying the criterion of a CDR score of ≥0.5 to each group: 14 of 32 subjects were identified in the PTSD group, 4 of 10 in the TBI group, 6 of 17 in the TBI+PTSD, and none of 21 in the healthy controls. Furthermore, MCI was diagnosed in 8 subjects in the PTSD group (6 amnestic and 2 nonamnestic), 4 subjects in the TBI group (all amnestic), and 3 subjects in the TBI+PTSD group (all amnestic; Table 1). Thus, we found that at least one third of the subjects with PTSD and/or TBI had a significant memory decline based on the CDR score, most of whom were diagnosed with amnestic MCI, suggesting an ongoing memory decline with likely eventual conversion to AD. Those same subjects had tau pathology with Braak stages II–V, which is consistent with previously reported findings in MCI subjects [22].

### Differences in tau deposition between TBI and/or PTSD groups

**Fig. 2:**
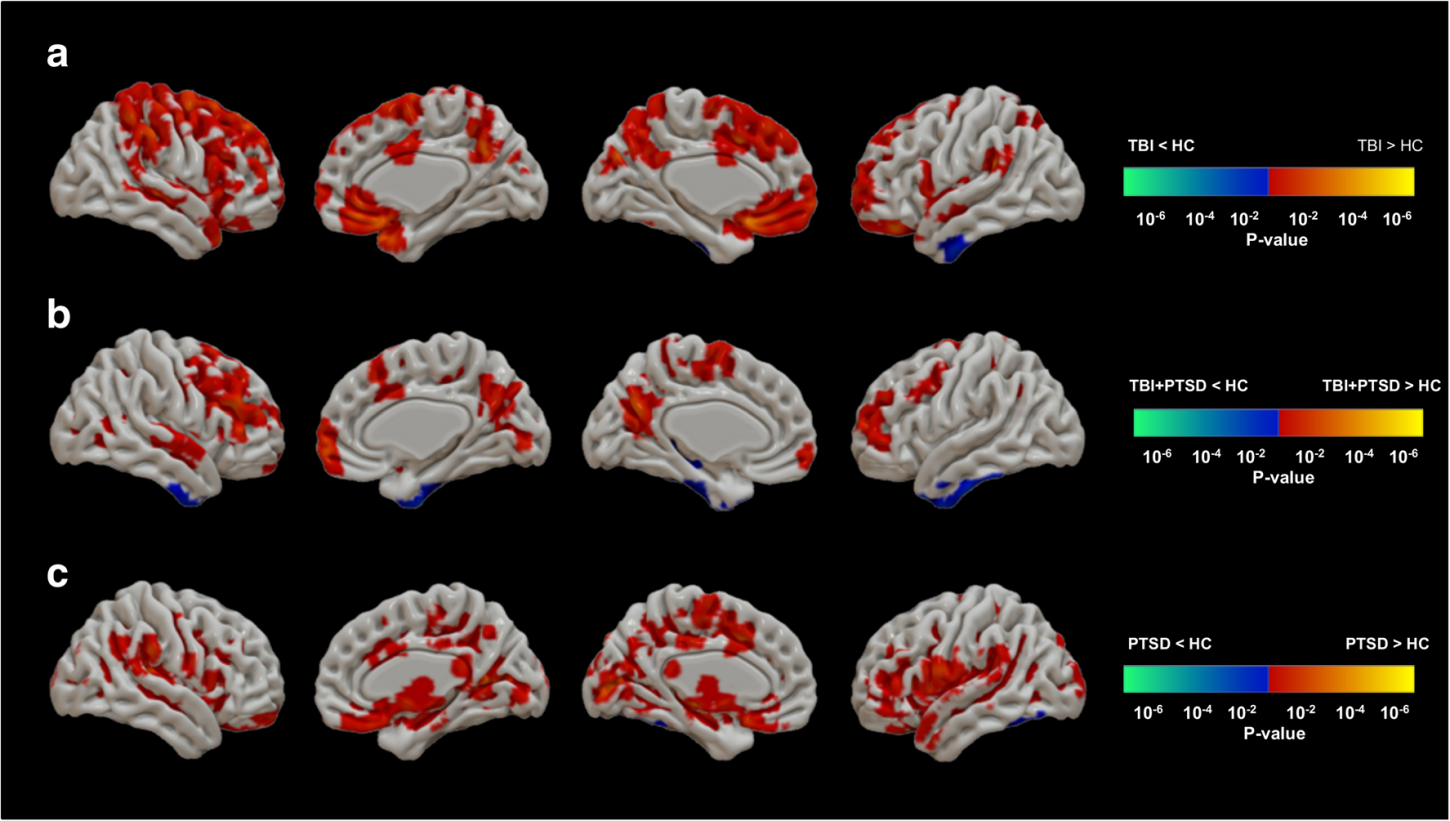
Comparisons of [^18^F]AV1451 SUVr between healthy controls and (**a**) the TBI group, (**b**) the TBI+PTSD group, and (**c**) the PTSD group. The *red–yellow* scale represents more tau accumulation in the clinical groups and the *blue–green* scale indicates more tau in healthy controls. Difference maps were calculated using the unpaired *t* test for [^18^F]AV1451 uptake in the clinical groups. The results were corrected using the false discovery rate (*p* < 0.05, cluster volume >40 voxels). *TBI* traumatic brain injury, *PTSD* posttraumatic stress disorder, *TBI+PTSD* TBI subjects who developed PTSD

Foci of mean [^18^F]AV1451 SUVr that showed significant differences between healthy controls and each of the three clinical groups are shown in Fig. 2. The TBI group (Fig. 2a) showed significantly higher mean SUVr in the superior frontal gyrus (SFG; 1.17 ± 0.05 versus 1.12 ± 0.10; *p* = 0.01), middle frontal gyrus (MFG; 1.18 ± 0.12 versus 1.16 ± 0.12; *p* = 0.02), medial orbitofrontal cortex (mOFC; 1.21 ± 0.14 versus 1.16 ± 0.13; *p* = 0.001), lateral orbitofrontal cortex (OFC; 1.18 ± 0.12 versus 1.16 ± 0.12; *p* = 0.01), precentral gyrus (1.19 ± 0.14 versus 1.16 ± 0.13; *p* = 0.001), postcentral gyrus (1.10 ± 0.06 versus 0.99 ± 0.07; *p* = 0.025), insula (1.13 ± 0.17 versus 1.10 ± 0.11; *p* = 0.003), supramarginal gyrus (SMG; 1.08 ± 0.08 versus 1.00 ± 0.07; *p* = 0.015), precuneus (1.11 ± 0.08 versus 1.06 ± 0.07; *p* = 0.03), STG (1.17 ± 0.12 versus 1.03 ± 0.11; *p* = 0.04), transverse temporal gyrus (TTG; 1.10 ± 0.11 versus 0.93 ± 0.06; *p* = 0.02), temporal pole (1.10 ± 0.07 versus 1.04 ± 0.11; *p* = 0.04), STG (1.08 ± 0.08 versus 1.00 ± 0.07; *p* = 0.003), and basal ganglia (1.41 ± 0.16 versus 1.32 ± 0.15; *p* = 0.05). Tau PET SUVr was lower in the TBI group compared to the control group in the left inferior temporal gyrus (ITG; 1.21 ± 0.16 versus 1.25 ± 0.15; *p* = 0.04).

The TBI+PTSD group (Fig. 2b) showed higher mean [^18^F]AV1451 SUVr in the TTG (1.10 ± 0.08 versus 0.93 ± 0.06; *p* = 0.045), SMG (1.10 ± 0.17 versus 1.01 ± 0.10; *p* = 0.032), MFG (1.04 ± 0.19 versus 0.97 ± 0.1; *p* = 0.05), precuneus (1.13 ± 0.2 versus 1.06 ± 0.07; *p* = 0.04), and STG (1.06 ± 0.13 versus 1.00 ± 0.07; *p* = 0.05. The PTSD group (Fig. 2c) showed higher mean SUVr in the brainstem (1.00 ± 0.01 versus 0.95 ± 0.05; *p* = 0.03), precuneus (1.1 ± 0.07 versus 1.06 ± 0.07; *p* = 0.05), insula (1.07 ± 0.09 versus 1.03 ± 0.06; *p* = 0.02), pars-opercularis (1.04 ± 0.1 versus 1.0 ± 0.06; *p* = 0.04), cuneus (1.10 ± 0.09 versus 1.05 ± 0.06; *p* = 0.02), pericalcarine (1.10 ± 0.1 versus 1.07 ± 0.07; *p* = 0.03), STG (1.04 ± 0.07 versus 0.99 ± 0.07; *p* = 0.05), TTG (1.00 ± 0.08 versus 0.93 ± 0.06; *p* = 0.003), mOFC (1.07 ± 0.07 versus 1.02 ± 0.07; *p* = 0.04). [^18^F]AV1451 SUVr in the ITG was lower in the TBI group (1.18 ± 0.17 versus 1.25 ± 0.15; *p* = 0.04) than in healthy controls.

**Fig. 3:**
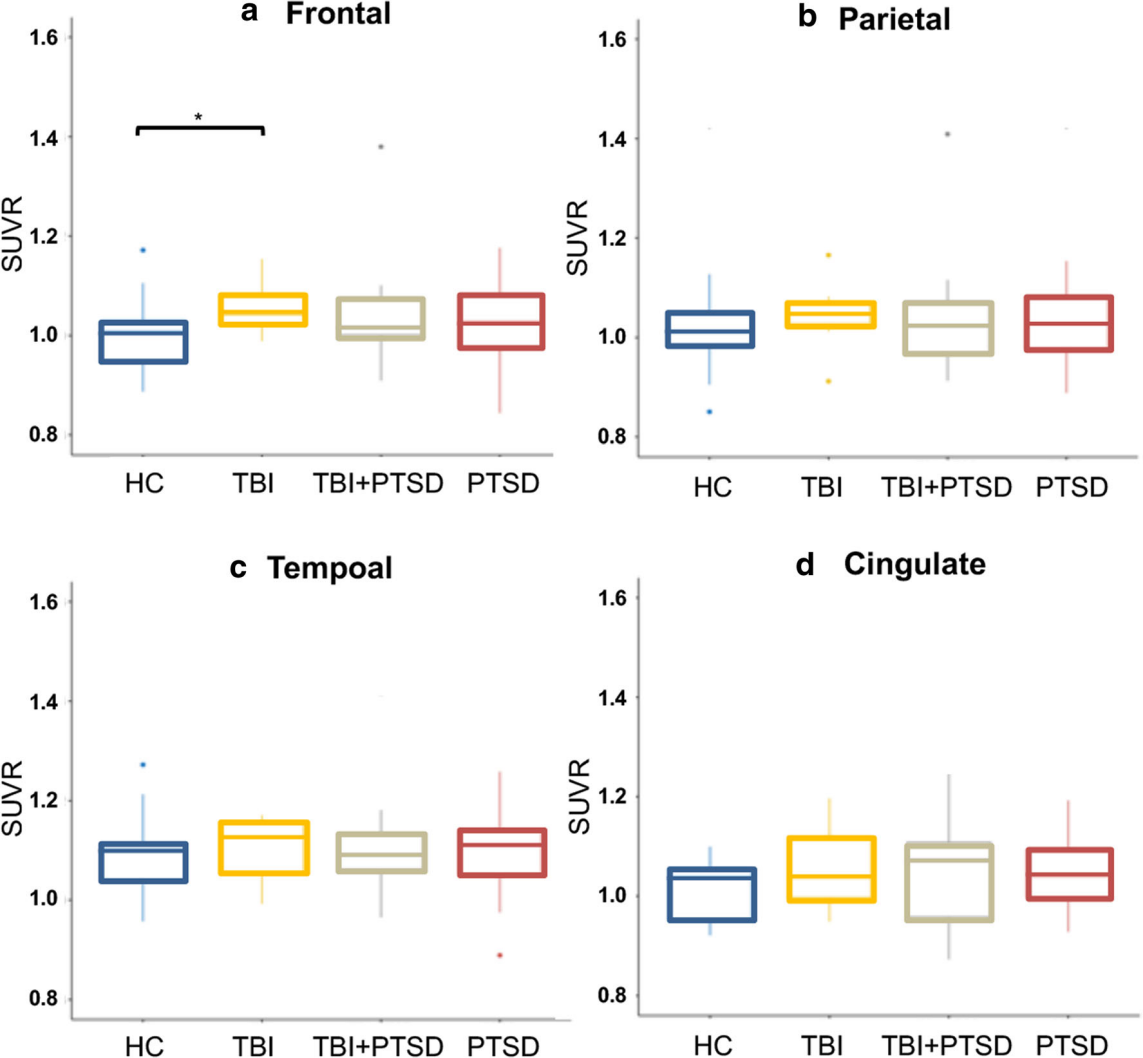
[^18^F]AV1451 SUVr in four cortical regions of interest including (**a**) the frontal cortex, (**b**) the parietal cortex, (**c**) the temporal cortex, and (**d**) cingulate cortex. *TBI* traumatic brain injury, *PTSD* posttraumatic stress disorder, *TBI+PTSD* TBI subjects who developed PTSD

Figure 3 shows the [^18^F]AV1451 SUVr in the frontal, parietal, and temporal lobes along with the cingulate cortex in each of the clinical groups. The TBI group showed significantly higher SUVr only in the frontal lobe (1.06 ± 0.05 versus 1.00 ± 0.07; *p* = 0.015) as compared to healthy controls, whereas the PTSD and TBI+PTSD groups showed no significant differences in any of the large ROIs compared to healthy controls (*p* > 0.05).

### Correlation between regional [^18^F]AV1451 SUVr and clinical outcomes

**Fig. 4:**
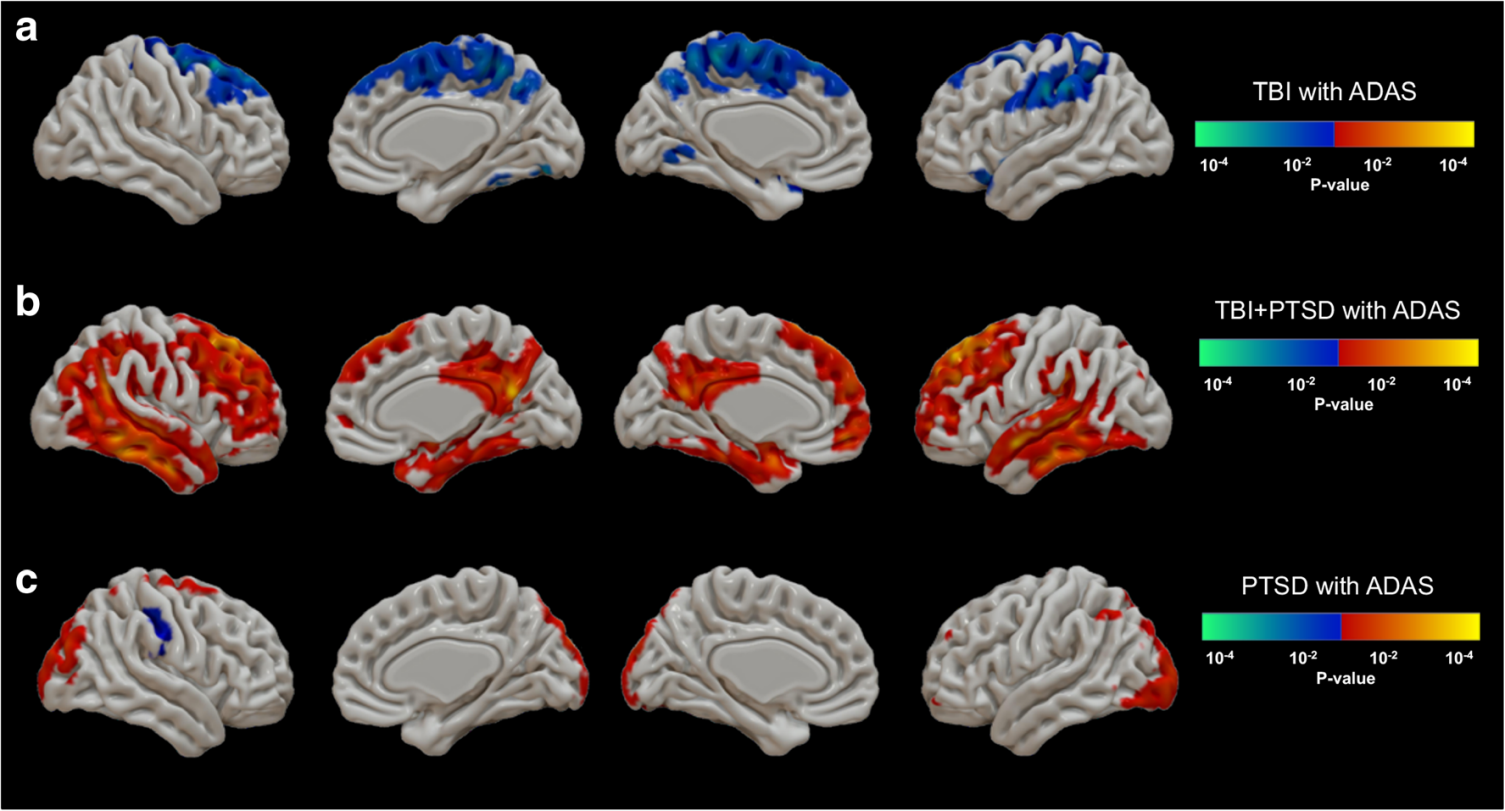
Correlations between [^18^F]AV1451 SUVr and ADAS-Cog scores in (**a**) the TBI group, (**b**) the TBI+PTSD group, and (**c**) the PTSD group. The *red–yellow* scale represents positive correlations between [^18^F]AV1451 SUVr and the ADAS-Cog score, while the *blue–green* scale represents negative correlations between [^18^F]AV1451 SUVr and the ADAS-Cog score. *TBI* traumatic brain injury, *PTSD* posttraumatic stress disorder, *TBI+PTSD* TBI subjects who developed PTSD

The TBI group (Fig. 4a) showed negative correlations between the ADAS-Cog score and [^18^F]AV1451 SUVr in the anterior cingulate cortex (ACC; *r* = −0.65; *p* = 0.04), paracentral gyrus (*r* = −0.71; *p* = 0.02), and SFG (*r* = −0.45; *p* = 0.02). The TBI+PTSD group (Fig. 4b) showed significant positive correlations between the ADAS-Cog score and [^18^F]AV1451 SUVr in the frontal pole (*r* = 0.59; *p* = 0.01), posterior cingulate cortex (PCC; *r* = 0.53; *p* = 0.03), SFG (*r* = 0.69; *p* < 0.01), pars-orbital gyrus (*r* = 0.59; *p* = 0.01), pars-triangularis (*r* = 0.7; *p* < 0.01), pars-opercularis (*r* = 0.63; *p* = 0.01), rostral MFG (*r* = 0.71; *p* < 0.01), temporal pole (*r* = 0.51; *p* = 0.04), transentorhinal cortex (*r* = 0.55; *p* = 0.02), MTG (*r* = 0.71; *p* < 0.01), ITG (*r* = 0.72; *p* < 0.01), STG (*r* = 0.69; *p* < 0.01), lateral occipital cortex (*r* = 0.58; *p* = 0.01), fusiform gyrus (*r* = 0.62; *p* = 0.01), inferior parietal lobule (IPL; *r* = 0.67; *p* < 0.01), superior parietal lobule (SPL; *r* = 0.66; *p* < 0.01), and SMG (*r* = 0.72; *p* < 0.01), while in the PTSD group (Fig. 4c) a positive correlation was observed in the lateral occipital cortex (*r* = 0.43; *p* = 0.05).

**Fig. 5:**
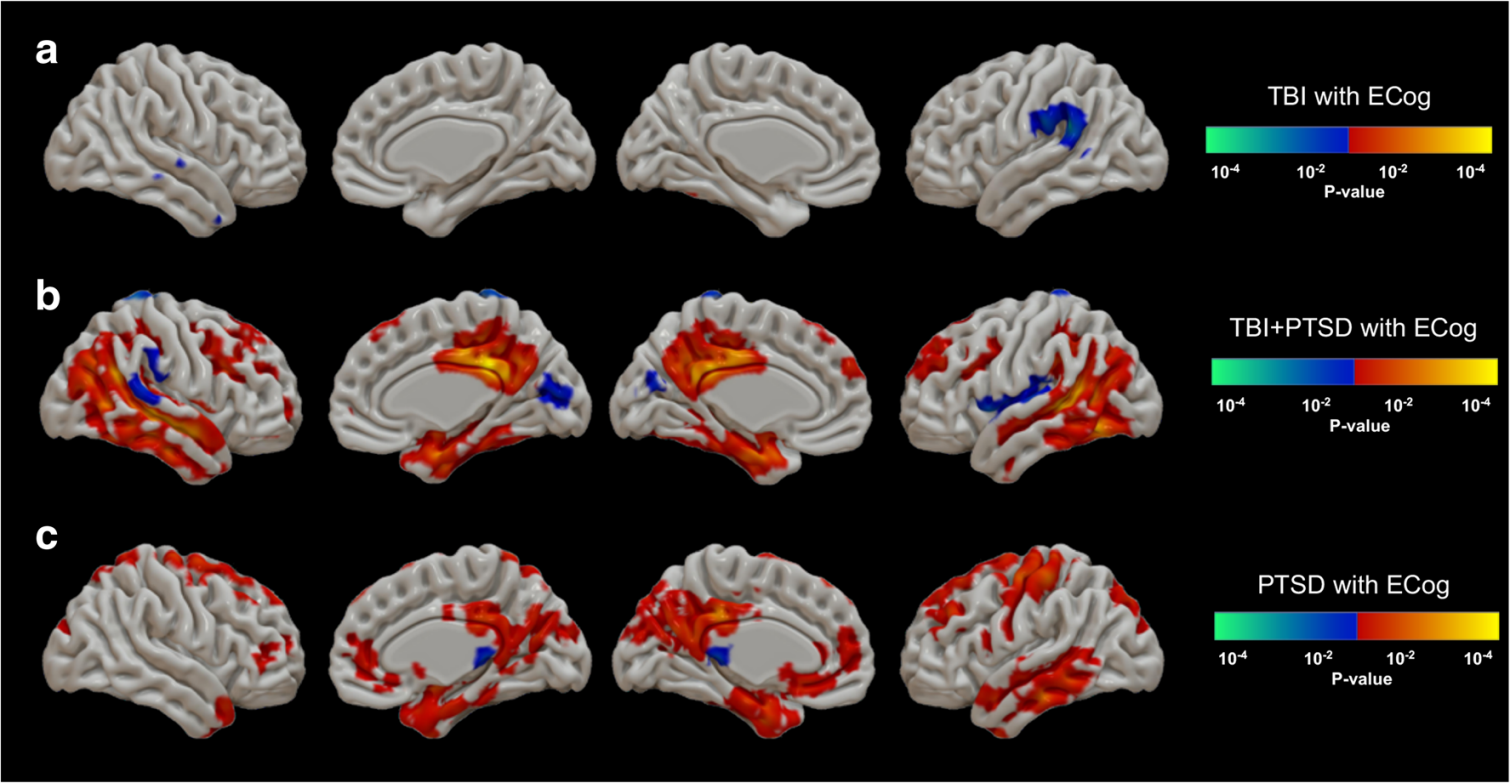
Correlations between [^18^F]AV1451 SUVr and the ECog total score in (**a**) the TBI group , (**b**) the TBI+PTSD group, and (**c**) the PTSD group. The *red–yellow* scale represents positive correlations between [^18^F]AV1451 SUVr in the clinical groups and the ECog score, while the *blue–green* scale represents negative correlation between [^18^F]AV1451 SUVr and the ECog score. *TBI* traumatic brain injury, *PTSD* posttraumatic stress disorder, *TBI+PTSD* TBI subjects who developed PTSD

The TBI group (Fig. 5a) showed a negative correlation between [^18^F]AV1451 SUVr and ECog total score only in the left SMG (*r* = −0.45; *p* = 0.02), while the TBI+PTSD group (Fig. 5b) showed positive correlations in the ACC (*r* = 0.59; *p* = 0.01), PCC (*r* = 0.64; *p* < 0.01), lateral oribito-frontal gyrus (OFG; *r* = 0.52; *p* = 0.03), SFG (*r* = 0.69; *p* < 0.01), MFG (*r* = 0.62; *p* = 0.01), MTG (*r* = 0.6; *p* = 0.01), parahippocampus (*r* = 0.59; *p* = 0.01), temporal pole (*r* = 0.58; *p* = 0.01), fusiform gyrus (*r* = 0.7; *p* < 0.01), ITG (*r* = 0.69; *p* < 0.1), STG (*r* = 0.65; *p* < 0.01), transentorhinal cortex (*r* = 0.8; *p* < 0.01), precuneus (*r* = 0.56; *p* = 0.02), IPL (*r* = 0.65; *p* = 0.01), SPL (*r* = 0.68; *p* < 0.01), supramarginal gyrus (*r* = 0.70; *p* < 0.01), and amygdala (*r* = 0.63; *p* = 0.01), and negative correlations in the cuneus (*r* = −0.47; *p* = 0.01), SMG (*r* = −0.47; *p* = 0.04), and left insula (*r* = −0.50; *p* = 0.02). The PTSD group (Fig. 5c) showed positive correlations in the caudal MFG (*r* = 0.4; *p* = 0.02), fusiform gyrus (*r* = 0.35; *p* = 0.05), lateral occipital cortex (*r* = 0.40; *p* = 0.02), ITG (*r* = 0.40; *p* = 0.02), postcentral gyrus (*r* = 0.40; *p* = 0.02), precentral gyrus (*r* = 0.41; *p* = 0.02), PCC (*r* = 0.38; *p* = 0.03), ACC (*r* = 0.45; *p* = 0.01), SPL (*r* = 0.41; *p* = 0.02), and cuneus (*r* = 0.47; *p* = 0.01).

**Fig. 6:**
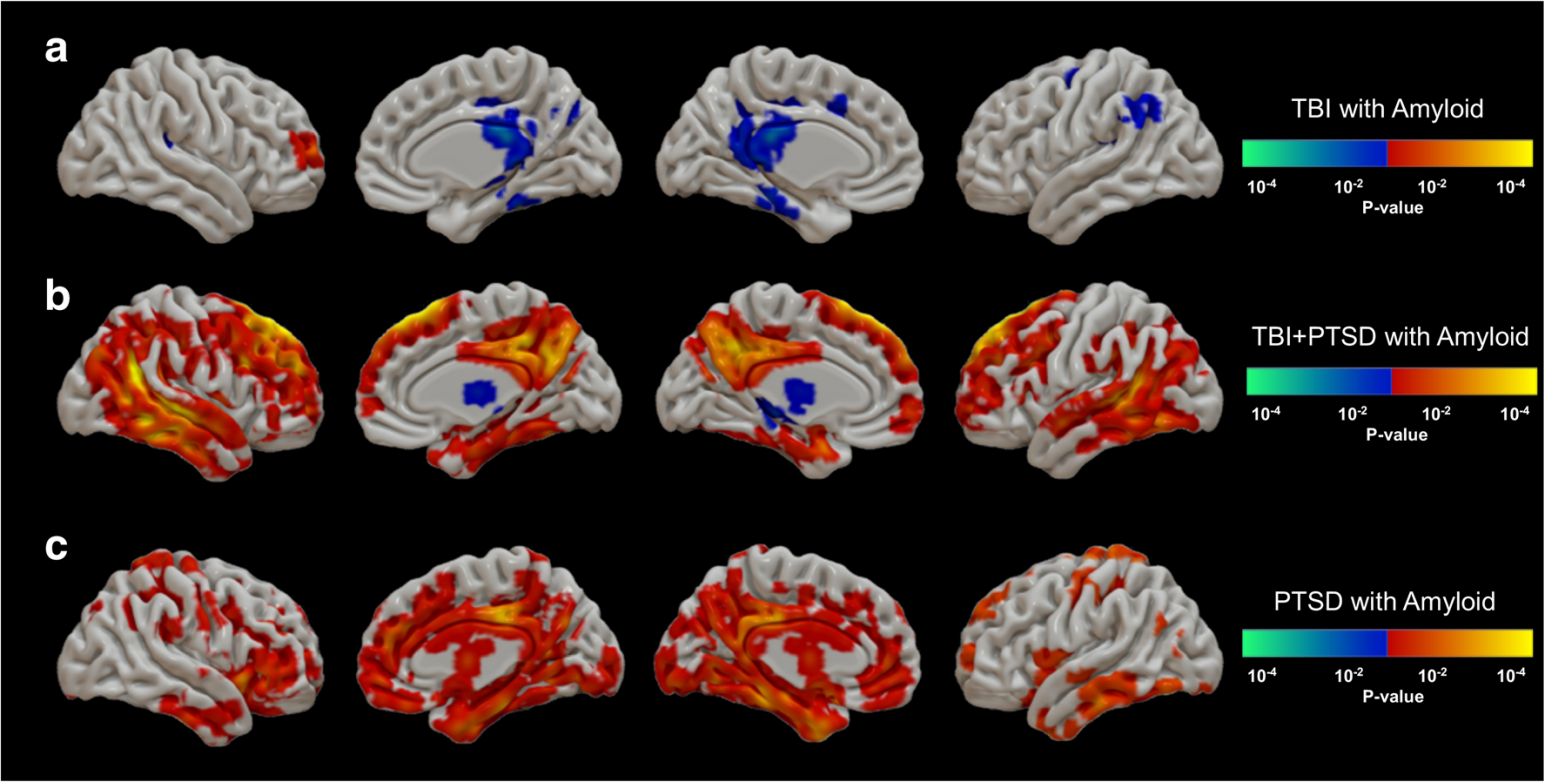
Correlations between [^18^F]AV1451 SUVr and the total cortical amyloid represented by mean [^18^F]AV45 SUVr in (**a**) the TBI group, (**b**) the TBI+PTSD group, and (**c**) the PTSD group. The *red–yellow* scale represents positive correlations between tau and amyloid accumulation in the clinical groups, while the *blue–green* scale represents negative correlations between tau and amyloid accumulation. *TBI* traumatic brain injury, *PTSD* posttraumatic stress disorder, *TBI+PTSD* TBI subjects who developed PTSD

Figure 6 shows the correlations between total cortical amyloid reprsented by mean [^18^F]AV45 SUVr and [^18^F]AV1451 SUVr voxel-wise maps. The TBI group (Fig. 6a) showed negative correlations in the PCC (*r* = −0.56; *p* = 0.02), SMG (*r* = 0.50; *p* = 0.04), and transentorhinal cortex (*r* = 0.46; *p* < 0.05), and a positive correlation in the MFG (*r* = 0.40; *p* < 0.05). The TBI+PTSD group (Fig. 6b) showed positive correlations in the PCC (*r* = 0.64; *p* = 0.01), pars-opercularis (*r* = 0.69; *p* < 0.01), MFG (*r* = 0.73; *p* < 0.01), frontal pole (*r* = 0.84; *p* < 0.01), SFG (*r* = 0.75; *p* < 0.01), precuneus (*r* = 0.66; *p* < 0.01), IPL (*r* = 0.79; *p* < 0.01), SPL (*r* = 0.73; *p* < 0.01), SMG (*r* = 0.80; *p* < 0.01), transentorhinal cortex (*r* = 0.57; *p* = 0.02), fusiform gyrus (*r* = 0.71; *p* < 0.01), temporal pole (*r* = 0.66; *p* < 0.01), ITG (*r* = 0.83; *p* < 0.01), MTG (*r* = 0.86; *p* < 0.1), and STG (*r* = 0.81; *p* < 0.01), while negative correlations were observed in the caudate nucleus (*r* = −0.54; *p* = 0.02), pallidum (*r* = −0.49; *p* = 0.04), and thalamus (*r* = −0.62; *p* = 0.01). The PTSD group (Fig. 6c) showed positive correlations in the amygdala (*r* = 0.38; *p* = 0.03), fusiform gyrus (*r* = 0.38; *p* = 0.03), hippocampus (*r* = 0.36; *p* = 0.04), ITG (*r* = 0.36; *p* = 0.04), MTG (*r* = 0.37; *p* = 0.04), parahippocampus (*r* = 0.5; *p* < 0.01), transentorhinal cortex (*r* = 0.45; *p* = 0.01), ACC (*r* = 0.38; *p* = 0.03), and PCC (*r* = 0.50; *p* < 0.01).

Of the three clinical groups, the TBI+PTSD group showed the most significant positive correlations between tau PET data and CDR scores, whereas the PTSD group showed a trend towards a positive correlation (see Supplementary Fig. 1). This might suggest that individuals with a CDR score ≥0.5 have relatively more tau accumulation in regions typically involved in AD. However, as part of the inclusion criteria of ADNI-DOD, none of the participants had a diagnosis of AD or other dementia at the time of scanning.

### Estimated Braak stages for different clinical groups recapitulated by [^18^F]AV1451 PET

The [^18^F]AV1451 PET images in the present study exhibited distributions of tau pathology consistent with those expected from post-mortem studies of healthy control, MCI and AD subjects. Many subjects (mostly healthy controls) showed a uniformly low cortical SUVr similar to the reference region’s SUVr (stage 0; *n* = 39), while other subjects showed focally increased tracer retention in the medial temporal lobes consistent with Braak stages I–III (*n* = 35), and still others showed more widespread distributions of tracer binding characteristic of Braak stage IV (*n* = 5) and even Braak stage V (*n* = 1; Table 2). Upon further visual investigation, subjects in the TBI group exhibited increased SUVr in the frontal cortex in association with the different Braak stages shown in Table 2.

**Table 2.** Estimated Braak stages in different groups

Braak stage	Group (*n*)								
	Total	Healthy controls		TBI		TBI+PTSD		PTSD	
		Amyloid-negative	Amyloid-positive	Amyloid-negative	Amyloid-positive	Amyloid-negative	Amyloid-positive	Amyloid-negative	Amyloid-positive
0	39	10	4	1	2	4	4	7	7
I	3	0	0	1	0	0	0	2	0
II	25	3	3	1	2	3	3	5	5
III	7	0	0	0	2	1	1	0	3
IV	5	0	1	0	1	0	0	0	3
V	1	0	0	0	0	0	1	0	0
VI	0	0	0	0	0	0	0	0	0
Total	80	13	8	3	7	8	9	14	18

*TBI* traumatic brain injury, *PTSD* posttraumatic stress disorder, *TBI+PTSD* subjects with TBI who developed PTSD

## Discussion

In vivo tau PET imaging in our clinical groups revealed increased tau tracer binding with topographical patterns resembling the distributions of tau pathology in neurodegenerative disorders such as AD and CTE [29–32]. We also observed positive correlations between tau and the severity of deficits in the various cognitive tests in the PTSD and TBI+PTSD groups. These results suggest that a history of TBI and/or PTSD might initiate pathological changes eventually coming to resemble aspects of tauopathy in AD, and manifesting in significant (but not yet pathological) deficits across a range of cognitive domains.

### Neurocognition suggests more progressive impairments of TBI+PTSD and PTSD towards AD

Among subjects in the investigated clinical groups, PTSD subjects exhibited the worst cognitive performance in all assessments, followed by TBI+PTSD subjects, whereas cognitive scores in TBI subjects and healthy controls did not differ significantly (Fig. 1, Table 1). Yaffe et al. have shown that military personnel with PTSD are twice as likely to develop dementia as those without PTSD [10]. TBI and PTSD are highly comorbid conditions in civilian life and among veterans [7, 9], and both conditions are associated with an increased risk of developing dementia later in life [5]. This link between TBI and PTSD may result from the physical injury and consequent cognitive impairments arising from TBI [33], or may be due to persistent trauma-related memory [34]. The ADAS-Cog, MMSE, ECog and CDR scores all showed greater memory and cognitive impairment in subjects with PTSD, recapitulating the findings of our earlier study in a larger group of ADNI-DOD subjects who had undergone amyloid PET imaging [25]. In their review, Regehr and LeBlanc found that the degree of impairment of cognitive and working memory was correlated with the severity of PTSD [35].

In the present study, >35% of the subjects with TBI and/or PTSD had some memory decline (CDR score ≥0.5), and most of these subjects were diagnosed with amnestic MCI, suggesting a progressive memory decline and raising the suspicion of early AD pathology. Indeed, these subjects were classified as Braak stages II–V, which is consistent with the range of Braak stages reported in MCI subjects [22]. Furthermore, this also suggests that a history of TBI and/or PTSD might predict memory deficits occurring decades after the trauma.

### Increased tau deposition might suggest typical AD progression in TBI+PTSD and PTSD as a possible link to AD

In the present study, elevated tau deposition (10–20%) was found in the cerebral cortex of TBI subjects compared with controls. Tau is a scaffolding protein binding axonal microtubules and other proteins, and TBI causes tau to abnormally phosphorylate, misfold and cleave, and thus to form NFTs [36]. A post-mortem study of long-term (up to 49 years) survivors of a single TBI event showed exceptionally abundant NFTs in the cingulate gyrus, SFG and insular cortex, which led the authors to suggest a causal relationship between a single TBI event and the acquisition of AD-like neuropathological features [15]. Tauopathy has also been reported in cohorts of individuals with a history of repetitive TBI leading to CTE and ultimately proceeding to AD [16, 32], and in a group of players of American football with repeated concussion who showed high [^18^F]AV1451 uptake in the cortical grey matter–white matter junction of multiple regions, which is considered pathognomonic for CTE [16]. The relationship between TBI and tau deposition may be a consequence of the physical damage to the axonal cytoskeleton by shearing forces [37] in conjunction with the nucleation of abnormal tau promoting the formation of NFTs [38]. This biophysical model of tau pathogenesis was proposed by Ahmadzadeh et al., who suggested that tau-crosslinked microtubules are sufficiently flexible to accommodate mechanical strain in the brain when it arises slowly [39, 40], but may fail if severe mechanical strain arises rapidly, and thus overwhelms the integrity of microtubules crosslinked by tau, causing tau dissociation and aggregation [41, 42].

Another possible mechanism may be that damage to the blood–brain barrier (BBB) after TBI facilitates tau accumulation. In this scenario, TBI induces NFT formation particularly around small blood vessels of the cortex, typically in the depths of the sulci, and this may lead to CTE [32]. Ramos-Cejudo et al. proposed that TBI first accelerates amyloid aggregation, leading to cerebrovascular injury and BBB damage, which then results in a deleterious feed-forward mechanism in which increased arterial stiffness favours further amyloid and tau deposition [42]. PET and histopathological examination have shown that amyloid plaque density increased within a year of the occurrence of a TBI event [43, 44]. On the other hand, Chen et al. found no evidence of provoked amyloid plaques in subjects who had suffered their TBI 3 years previously, despite ongoing elevation of the expression of the amyloid precursor protein in the white matter [45].

Taken together, these studies imply that transient amyloid plaques may form rapidly after TBI, but are normally cleared in subsequent years. This acute or transient response to TBI might be an initiator of a more chronic increase in tau accumulation in a pathological cascade that eventually leads to a form of tauopathy. The TBI group included subjects showing an AD-typical profile of tau deposition, with regions of increased tau appearing during Braak stages I–IV, in addition to atypical-AD regions including the frontal and cingulate cortex (Fig. 2a). A sea change in the perception of the long-term consequences of TBI has been seen in recent years, suggesting that the risks of CTE, Lewy body disease and parkinsonism are higher than the risk of AD [46–48]. However, we cannot currently establish if the increased tau in our TBI group was related to AD per se or to other tauopathies, mainly because of the absence of most of the cognitive impairments evident in the PTSD groups. Longitudinal tau PET studies in this or a similar cohort may better establish the relationship between TBI and AD-like pathology.

The PTSD group also showed elevated tau accumulation in the neocortex compared with controls. A single report has so far shown increased binding of the tau tracer [^18^F]AV1451 in subjects with chronic PTSD from an Australian cohort of Vietnam War veterans [19]. To elucidate the underlying mechanism by which PTSD induces tau accumulation, Miller et al. investigated the influence of the lipoxygenase genes *ALOX12* and *ALOX15* (enzymes involved in inflammatory responses) on the decreasing cerebrocortical thickness seen in subjects with PTSD, and found that *ALOX12* moderates the association between PTSD severity and thinning of the prefrontal cortex [49]. The *ALOX12* pathway has been found to modulate tau metabolism [50] and may be a mediator of inflammatory mechanisms in early AD [51].

By examining the tau accumulation profiles in individual subjects, we were able to identify those with PTSD and TBI+PTSD who showed similar tau profiles to that in AD patients. Jack proposed that early accumulation of cortical amyloid might accelerate the progression and spread of tauopathy in AD [52]. This author proposed that “primary age-related tauopathy” develops at some stage in life followed by increased amyloid deposition in certain neocortical areas that triggers (by an unidentified mechanism) accelerated tauopathy ultimately leading to severe cognitive deficits and AD [20, 22–24]. In the current study, elevated tau binding on PET was positively correlated with amyloid positivity and cognitive impairment in the PTSD and TBI+PTSD groups, but this association was not present in the TBI or healthy control groups, suggesting a particular association with PTSD.

Although none of the participants in our cohorts met the clinical diagnosis of AD, the correlation analysis of amyloid and tau PET findings suggested a strong predisposition for tau accumulation to track amyloid deposition, especially in the TBI+PTSD group, thus suggesting a complex relationship between the two pathologies. However, further investigation is required to substantiate this association. In amyloid-negative subjects, tauopathy with Braak stages above zero might be primary age-related tauopathy [37], and this also might explain the occasional finding of tau accumulation in our healthy control group. Alternately, our criterion for amyloid PET positivity of SUVr >1.1 in the whole cerebral cortex [53] may have resulted in early amyloid changes being missed in some subjects.

We found significant correlations between ADAS-Cog, ECog total and CDR scores and tau accumulation in both the TBI+PTSD and PTSD groups, with the most compelling correlations in the TBI+PTSD group (Figs. 4 and 5, and Supplementary Fig. 1). In this group, the spatial pattern of positive correlations broadly matched the default mode network (DMN), that involves the precuneus, PCC and medial frontal cortex [54]. Furthermore, tau accumulation in these same regions was positively correlated with total cortical amyloid deposition (Fig. 6). These regions of the DMN have previously been shown to contain amyloid deposits in patients with MCI [55] and early AD [54], suggesting that the DMN is the first functional network to be disrupted in AD [55]. These various correlations between tau and cognitive impairments and amyloid may suggest that TBI+PTSD and PTSD subjects are at higher risk of conversion to AD, following the typical AD progression profile proposed by the NIA-AA framework [20].

The data presented here imply that those veterans who developed PTSD following their TBI might be at the highest risk of progression to AD, while those with TBI only might be more at risk of developing other neuropathies [46–48], a conjecture that could be investigated by longitudinal molecular imaging studies. Work by Li et al. showed that a self-reported history of TBI was associated with an onset of cognitive impairment in older adults 3–4 years earlier than in those without a history of TBI [56], but these authors did not report interactions with PTSD.

The major limitation of this study was the small number of subjects in the TBI cohort (*n* = 10), which was insufficient to support strong conclusions. Further investigations are required to establish better links between TBI or PTSD with tau pathology and the risk of AD or other forms of dementia. In addition, there is a need for further investigation of the mechanisms triggering AD onset and progression. Future studies in a larger cohort may establish cut-off criteria for tau PET conforming to Braak staging.

### Conclusion

Our findings show for the first time that a history of TBI and/or PTSD is associated with increased tauopathy resembling AD-typical and atypical patterns, and is correlated with impaired neuropsychological function relative to healthy controls.

## Electronic supplementary material

Supplementary Fig. 1 Correlation between [^18^F]AV1451 SUVr maps and clinical dementia rating (CDR) score in (**a**) the TBI group, (**b**) the TBI+PTSD group, and (**c**) the PTSD group. The *red–yellow* scale represents positive correlations between tau accumulation in the clinical groups and the CDR score, while the *blue–green* scale represents negative correlations between tau and amyloid accumulation. *TBI* traumatic brain injury, *PTSD* posttraumatic stress disorder, *TBI+PTSD* TBI subjects who developed PTSD. (PNG 1161 kb)

High resolution image (TIF 9866 kb)

## References

[1] Daviglus ML, Plassman BL, Pirzada A, Bell CC, Bowen PE, Burke JR (2011). Risk factors and preventive interventions for Alzheimer disease: state of the science. Arch Neurol.

[2] Kerbler GM, Fripp J, Rowe CC, Villemagne VL, Salvado O, Rose S (2015). Basal forebrain atrophy correlates with amyloid β burden in Alzheimer’s disease. Neuroimage Clin.

[3] Weiner MW, Veitch DP, Aisen PS, Beckett LA, Cairns NJ, Green RC, et al. The Alzheimer's Disease Neuroimaging Initiative: a review of papers published since its inception. Alzheimer’s Dement. 2012;9:e111–94. 10.1016/j.jalz.2013.05.1769 PMC4108198 23932184

[4] Weiner MW, Veitch DP, Aisen PS, Beckett LA, Cairns NJ, Cedarbaum J, et al. 2014 Update of the Alzheimer's Disease Neuroimaging Initiative: a review of papers published since its inception. Alzheimers Dement. 2015;11:e1–120. 10.1016/j.jalz.2014.11.001 PMC5469297 26073027

[5] MacDonald CL, Barber J, Jordan M, Johnson AM, Dikmen S, Fann JR (2017). Early clinical predictors of 5-year outcome after concussive blast traumatic brain injury. JAMA Neurol.

[6] Fleminger S, Pondsford J (2005). Long term outcome after traumatic brain injury. BMJ.

[7] Alway Y, McKay A, Gould KR, Johnston L, Ponsford J (2016). Factors associated with posttraumatic stress disorder following moderate to severe traumatic brain injury: a prospective study. Depress Anxiety.

[8] Amen DG, Raji CA, Willeumier K, Taylor D, Tarzwell R, Newberg A (2015). Functional neuroimaging distinguishes posttraumatic stress disorder from traumatic brain injury in focused and large community datasets. PLoS One.

[9] Joshi S, Dunbar K, Taylor P, Sullivan KL, Afzal MM, Song C (2017). Streamlining participant recruitment for TBI and PTSD research studies. Mil Med.

[10] Yaffe K, Vittinghoff E, Lindquist K, Barnes D, Covinsky KE, Neylan T (2010). Posttraumatic stress disorder and risk of dementia among US veterans. Arch Gen Psychiatry.

[11] Fleminger S, Oliver DL, Lovestone S, Rabe-Hesketh S, Giora A (2003). Head injury as a risk factor for Alzheimer’s disease: the evidence 10 years on; a partial replication. J Neurol Neurosurg Psychiatry.

[12] Isoniemi H, Kurki T, Tenovuo O, Kairisto V, Portin R (2006). Hippocampal volume, brain atrophy, and APOE genotype after traumatic brain injury. Neurology.

[13] Lindauer RJL, Vlieger E-J, Jalink M, Olff M, Carlier I'VE, Majoie CBLM, et al. Effects of psychotherapy on hippocampal volume in out-patients with post-traumatic stress disorder: a MRI investigation. Psychol Med. 2005;35:1421–31. 10.1017/S0033291705005246 16164766

[14] van Rooij SJH, Kennis M, Sjouwerman R, van den Heuvel MP, Kahn RS, Geuze E (2015). Smaller hippocampal volume as a vulnerability factor for the persistence of post-traumatic stress disorder. Psychol Med.

[15] Johnson VE, Stewart W, Smith DH (2012). Widespread tau and amyloid-beta pathology many years after a single traumatic brain injury in humans. Brain Pathol.

[16] Dickstein DL, Pullman MY, Fernandez C, Short JA, Kostakoglu L, Knesaurek K (2016). Cerebral [18F]T807/AV1451 retention pattern in clinically probable CTE resembles pathognomonic distribution of CTE tauopathy. Transl Psychiatry.

[17] Xia C-F, Arteaga J, Chen G, Gangadharmath U, Gomez LF, Kasi D (2013). [18F]T807, a novel tau positron emission tomography imaging agent for Alzheimer’s disease. Alzheimers Dement.

[18] Lemoine L, Gillberg PG, Svedberg M, Stepanov V, Jia Z, Huang J, et al. Comparative binding properties of the tau PET tracers THK5117, THK5351, PBB3, and T807 in postmortem Alzheimer brains. Alzheimer’s Res Ther. 2017;9:96. 10.1186/s13195-017-0325-z PMC5725799 29229003

[19] Cummins T, Elias A, Hopwood M, Rosenfeld J, DorÃ V, Lamb F (2016). Assessing Aβ & tau pathology in Vietnam war veterans with chronic Post-Traumatic Stress Disorder. J Nucl Med.

[20] Jack CR, Bennett DA, Blennow K, Carrillo MC, Dunn B, Haeberlein SB (2018). NIA-AA Research Framework: toward a biological definition of Alzheimer’s disease. Alzheimers Dement.

[21] Marquié M, Siao Tick Chong M, Antón-Fernández A, Verwer EE, Sáez-Calveras N, Meltzer AC (2017). [F-18]-AV-1451 binding correlates with postmortem neurofibrillary tangle Braak staging. Acta Neuropathol.

[22] Schwarz AJ, Yu P, Miller BB, Shcherbinin S, Dickson J, Navitsky M (2016). Regional profiles of the candidate tau PET ligand 18F-AV-1451 recapitulate key features of Braak histopathological stages. Brain.

[23] Cho H, Choi JY, Lee SH, Lee JH, Choi YC, Ryu YH (2017). Excessive tau accumulation in the parieto-occipital cortex characterizes early-onset Alzheimer’s disease. Neurobiol Aging.

[24] Phillips JS, Das SR, McMillan CT, Irwin DJ, Roll EE, Da Re F (2018). Tau PET imaging predicts cognition in atypical variants of Alzheimer’s disease. Hum Brain Mapp.

[25] Mohamed AZ, Cumming P, Srour H, Gunasena T, Uchida A, Haller CN (2018). Amyloid pathology fingerprint differentiates post-traumatic stress disorder and traumatic brain injury. Neuroimage Clin.

[26] Fischl B (2012). FreeSurfer. Neuroimage.

[27] Andersson JLR, Jenkinson M, Smith S. Non-linear registration aka spatial normalisation. FMRIB Technical Report TR07JA2. Oxford: FMRIB Centre; 2007. p. 21.

[28] Johnson KA, Schultz A, Betensky RA, Becker JA, Sepulcre J, Rentz D (2016). Tau positron emission tomographic imaging in aging and early Alzheimer disease. Ann Neurol.

[29] Braak H, Alafuzoff I, Arzberger T, Kretzschmar H, Tredici K (2006). Staging of Alzheimer disease-associated neurofibrillary pathology using paraffin sections and immunocytochemistry. Acta Neuropathol.

[30] Braak H, Braak E (1991). Neuropathological stageing of Alzheimer-related changes. Acta Neuropathol.

[31] Marquié M, Normandin MD, Vanderburg CR, Costantino IM, Bien EA, Rycyna LG (2015). Validating novel tau positron emission tomography tracer [F-18]-AV-1451 (T807) on postmortem brain tissue. Ann Neurol.

[32] Dekosky ST, Blennow K, Ikonomovic MD, Gandy S (2013). Acute and chronic traumatic encephalopathies: pathogenesis and biomarkers. Nat Rev Neurol.

[33] Xue C, Ge Y, Tang B, Liu Y, Kang P, Wang M (2015). A meta-analysis of risk factors for combat-related PTSD among military personnel and veterans. PLoS One.

[34] Klein E, Caspi Y, Gil S (2003). The relation between memory of the traumatic event and PTSD: evidence from studies of traumatic brain injury. Can J Psychiatry.

[35] Regehr C, Leblanc VR (2017). PTSD, acute stress, performance and decision-making in emergency service workers. J Am Acad Psychiatry Law.

[36] Polanco JC, Li C, Bodea LG, Martinez-Marmol R, Meunier FA, Götz J (2018). Amyloid-β and tau complexity – towards improved biomarkers and targeted therapies. Nat Rev Neurol.

[37] Crary JF, Trojanowski JQ, Schneider JA, Abisambra JF, Abner EL, Alafuzoff I (2014). Primary age-related tauopathy (PART): a common pathology associated with human aging. Acta Neuropathol.

[38] Li C, Götz J (2017). Somatodendritic accumulation of Tau in Alzheimer’s disease is promoted by Fyn-mediated local protein translation. EMBO J.

[39] Ahmadzadeh H, Smith DH, Shenoy VB (2015). Mechanical effects of dynamic binding between tau proteins on microtubules during axonal injury. Biophys J.

[40] Ahmadzadeh H, Smith DH, Shenoy VB (2014). Viscoelasticity of tau proteins leads to strain rate-dependent breaking of microtubules during axonal stretch injury: Predictions from a mathematical model. Biophys J.

[41] Kawata K, Liu CY, Merkel SF, Ramirez SH, Tierney RT, Langford D (2016). Blood biomarkers for brain injury: What are we measuring?. Neurosci Biobehav Rev.

[42] Ramos-Cejudo J, Wisniewski T, Marmar C, Zetterberg H, Blennow K, de Leon MJ (2018). Traumatic brain injury and Alzheimer’s disease: the cerebrovascular link. EBioMedicine.

[43] Ikonomovic MD, Buckley CJ, Heurling K, Sherwin P, Jones PA, Zanette M (2016). Post-mortem histopathology underlying β-amyloid PET imaging following flutemetamol F 18 injection. Acta Neuropathol Commun.

[44] Hong YT, Veenith T, Dewar D, Outtrim JG, Mani V, Williams C (2014). Amyloid imaging with carbon 11-labeled Pittsburgh compound B for traumatic brain injury. JAMA Neurol.

[45] Chen X-HH, Johnson VE, Uryu K, Trojanowski JQ, Smith DH (2009). A lack of amyloid β plaques despite persistent accumulation of amyloid β in axons of long-term survivors of traumatic brain injury. Brain Pathol.

[46] Weiner MW, Crane PK, Montine TJ, Bennett DA, Veitch DP (2017). Traumatic brain injury may not increase the risk of Alzheimer disease. Neurology.

[47] Crane PK, Gibbons LE, Dams-O’ Connor K, Trittschuh E, Leverenz JB, Dirk Keene C (2016). Association of traumatic brain injury with late-life neurodegenerative conditions and neuropathologic findings. JAMA Neurol.

[48] Nordstrom P, Michaëlsson K, Gustafson Y, Nordström A (2014). Traumatic brain injury and young onset dementia: a nationwide cohort study. Ann Neurol.

[49] Miller MW, Wolf EJ, Sadeh N, Logue M, Spielberg JM, Hayes JP (2015). A novel locus in the oxidative stress-related gene ALOX12 moderates the association between PTSD and thickness of the prefrontal cortex. Psychoneuroendocrinology.

[50] Giannopoulos PF, Joshi YB, Chu J, Praticò D (2013). The 12-15-lipoxygenase is a modulator of Alzheimer’s-related tau pathology in vivo. Aging Cell.

[51] Yao Y, Clark CM, Trojanowski JQ, Lee VM-Y, Praticò D (2005). Elevation of 12/15 lipoxygenase products in AD and mild cognitive impairment. Ann Neurol.

[52] Jack CR (2014). PART and SNAP. Acta Neuropathol.

[53] Clark CM, Pontecorvo MJ, Beach TG, Bedell BJ, Coleman RE, Doraiswamy PM (2012). Cerebral PET with florbetapir compared with neuropathology at autopsy for detection of neuritic amyloid-β plaques: a prospective cohort study. Lancet Neurol.

[54] Greicius MD, Srivastava G, Reiss AL, Menon V (2004). Default-mode network activity distinguishes Alzheimer’s disease from healthy aging: evidence from functional MRI. Proc Natl Acad Sci U S A.

[55] Palmqvist S, Schöll M, Strandberg O, Mattsson N, Stomrud E, Zetterberg H (2017). Earliest accumulation of β-amyloid occurs within the default-mode network and concurrently affects brain connectivity. Nat Commun.

[56] Li W, Risacher SL, McAllister TW, Saykin AJ (2016). Traumatic brain injury and age at onset of cognitive impairment in older adults. J Neurol.

