# Supplementary figures and images for: Tauopathy in veterans with long-term posttraumatic stress disorder and traumatic brain injury
Source: Eur J Nucl Med Mol Imaging. 2019 Jan 7;46(5):1139–51. doi: 10.1007/s00259-018-4241-7 (PMC6451714; doi:10.1007/s00259-018-4241-7)

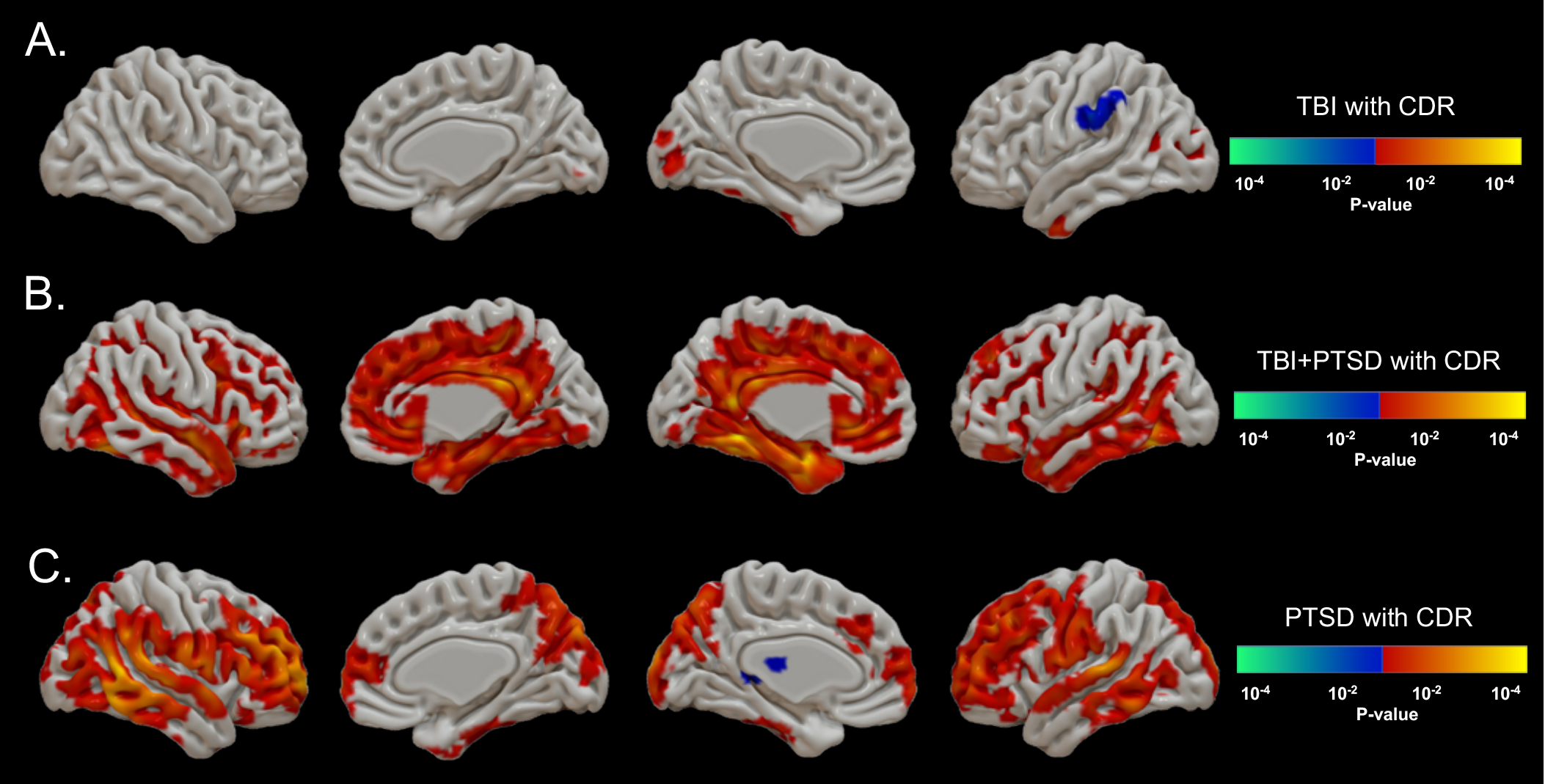

Supplement: Supplementary file 1 — Correlation between [18F]AV1451 SUVr maps and clinical dementia rating (CDR) score in (a) the TBI group, (b) the TBI+PTSD group, and (c) the PTSD group. The red–yellow scale represents positive correlations between tau accumulation in the clinical groups and the CDR score, while the blue–green scale represents negative correlations between tau and amyloid accumulation. TBI traumatic brain injury, PTSD posttraumatic stress disorder, TBI+PTSD TBI subjects who developed PTSD. (PNG 1161 kb) [file 259_2018_4241_Fig7_ESM.png]

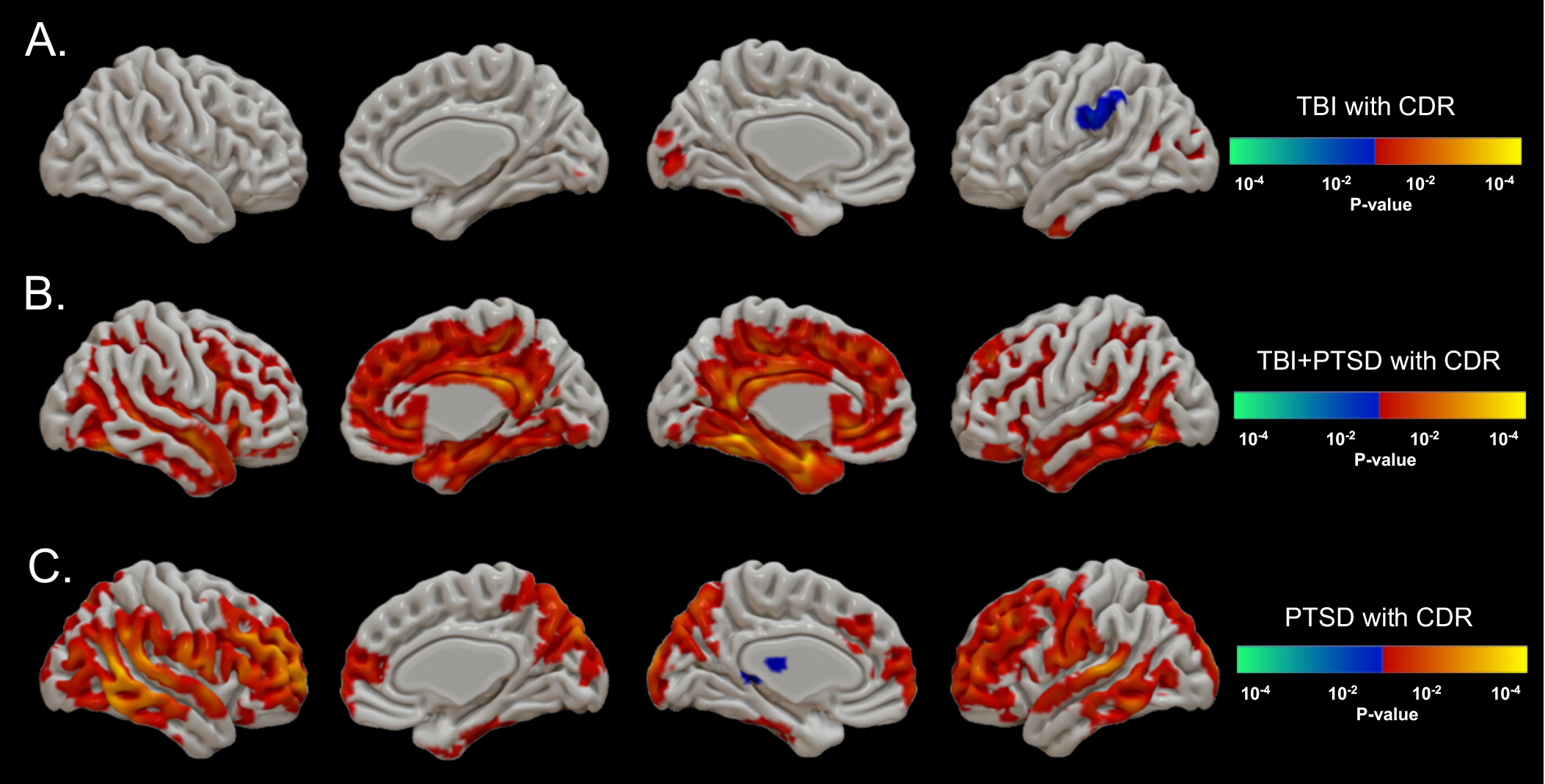

Supplement: Supplementary file 2 — High resolution image (TIF 9866 kb) [file 259_2018_4241_MOESM1_ESM.tif]
